# Analysis of lncRNA, miRNA, and mRNA Expression Profiling in Type I IFN and Type II IFN Overexpressed in Porcine Alveolar Macrophages

**DOI:** 10.1155/2021/6666160

**Published:** 2021-06-16

**Authors:** Congcong Li, Haoyuan Han, Xiuling Li, Jiao Wu, Xinfeng Li, Hui Niu, Wantao Li

**Affiliations:** ^1^College of Animal Science and Technology, Henan University of Animal Husbandry and Economy, Zhengzhou, Henan, China; ^2^College of Animal Science and Technology, Henan Agricultural University, Zhengzhou, Henan, China; ^3^Henan Key Laboratory of Unconventional Feed Resources Innovative Utilization, Henan University of Animal Husbandry and Economy, Zhengzhou, Henan, China

## Abstract

Current data is scarce regarding the function of noncoding RNAs (ncRNAs) such as microRNAs (miRNAs) and long noncoding RNAs (lncRNAs) in the interferon- (IFN-) mediated immune response. This is a comprehensive study that analyzes the lncRNA and miRNA expression profiles of the type I IFN and type II IFN in porcine alveolar macrophages using RNA sequencing. There was a total of 152 overexpressed differentially expressed (DE) lncRNAs and 21 DE miRNAs across type I IFN and type II IFN in porcine alveolar macrophages. Subsequent lncRNA-miRNA-mRNA network construction revealed the involvement of 36 DE lncRNAs and 12 DE miRNAs. LncRNAs such as the XLOC_211306, XLOC_100516, XLOC_00695, XLOC_149196, and XLOC_014459 were expressed at a higher degree in the type I IFN group, while XLOC_222640, XLOC_047290, XLOC_147777, XLOC_162298, XLOC_220210, and XLOC_165237 were expressed at a higher degree in the type II IFN group. These lncRNAs were found to act as “sponges” for miRNAs such as miR-34a, miR-328, miR-885-3p, miR-149, miR-30c-3p, miR-30b-5p, miR-708-5p, miR-193a-5p, miR-365-5p, and miR-7. Their target genes FADS2, RPS6KA1, PIM1, and NOD1 were found to be associated with several immune-related signaling pathways including the NOD-like receptor, Jak-STAT, mTOR, and PPAR signaling pathways. These experiments provide a comprehensive profile of overexpressed noncoding RNAs in porcine alveolar macrophages, providing new insights regarding the IFN-mediated immune response.

## 1. Introduction

Porcine products are important agricultural resources for the production of meat as well as a disease model for human medical research. Porcine products are also a source of nonhuman organs for human xenotransplantation [1]. The development of intensive feeding regimes in pig farming has led to the development of numerous viral and bacterial infectious diseases which may be detrimental to both animals and humans. Genetically modified porcine breeds were bred as a means to control the development and spread of swine-based diseases [2]. Molecular profile analysis of specific spatial and temporal immune-related phenotypes through transcriptome sequencing technology represents an important way in studying the genetic basis of disease resistance [3, 4].

Interferons (IFNs) were discovered in 1957 and were duly named as a tribute to their inherent capabilities in suppressing viral proliferation [5]. IFNs act as major regulators of the innate immune response to viral infections [6]. IFNs are also known to enhance or inhibit cellular differentiation, suppress cellular division, and are protective against parasitic and bacterial infections [7]. IFNs also play immunomodulatory roles. IFNs can inhibit B lymphocyte (B cell) activation, augment T lymphocyte (T cell) activity, and also enhance the cellular destructive ability of natural killer cells. Interferon alpha (*α*), beta (*β*), and gamma (*γ*) are three known forms of IFNs, which are further subcategorized in types I and II. Type I consists of IFN*α* and IFN*β*, while IFN*γ* represents a type II IFN. Type I IFN can be produced by all cells and is typically secreted after viral infections to induce cellular viral resistance. Type II IFNs are only produced by T lymphocytes and natural killer cells and trigger an immune response against cancer cells or infectious agents. As an antiviral agent, IFNs have an impact on both coding and noncoding genes regulating IFN. The cellular response to a viral infection is a combination of both IFNs and IFN-induced gene products.

The biological activity of IFN*α*/*β* and IFN*γ* is carried out through homologous cell receptor binding-mediated activation of signaling pathways such as the Janus-activated kinase/signal transducer and activator of the transcription (Jak/STAT) signaling pathway [8]. The IFN*α*/*β* receptor is composed of two main subunits IFNAR1 and IFNAR2 [9]. IFNGR1 and IFNGR2 are two polypeptides which are the cornerstones in forming the IFN-*γ* receptor [10]. Type I and type II IFNs stimulate the expressions of more than 2,000 IFN-enhancing genes (ISGs) which limit pathogen replication and enhance pathogen detection [11]. IFN signals are collectively regulated by environmental factors, the host, and the pathogen [12].

Although studies on IFN have traditionally focused on IFN itself or proteins induced by IFN, it is often overlooked that molecule also possesses a significant effect on both lncRNAs and miRNAs. Viral infections and IFNs are able to directly induce the miRNA expression. This has been exhibited in murine macrophages, where IFN*β* and poly (I: C) were able to induce the miR-155 expression [13]. Furthermore, miR-34a was found to be upregulated after RNA virus infection or IFN*β* induction. Conversely, the mRNA 5′cap-binding protein 4EHP was found to impede the IFN*β* expression [14]. In virus-infected host cells, viruses and miRNAs interact with IFN signaling pathways. It has been reported that PRRSV suppressed IFN*β* protein quantities in primary porcine alveolar macrophages (PAMs) during early infection. miRNAs including let-7b, miR-26a, miR-34a, and miR-145 were upregulated in PRRSV-infected PAMs. These same miRNAs were able to inhibit the IFN*β* protein expression in primary PAMs by directly targeting the 3′UTR of IFN*β* [15]. Lin et al. reported that the influenza A virus (IAV) utilizes a novel mechanism of stimulating host type I IFN-mediated antiviral immune responses by suppressing the expressions of miR-30 family members [16]. Interestingly, viruses which are able to encode miRNAs may directly alter the IFN response to infection. IFN*α*/*β*, which is homologous to the HIV genome 3′UTR, is known to induce miR-29a. miR-29a was able to reduce HIV replication, while inhibiting miR-29a enhanced viral replication. miR-29a-HIV mRNA was localized to endogenous RISC [17]. Additionally, IFN*γ* was able to upregulate miR-29 expressions through the STAT-1 signaling pathway [18]. IFN*β* also induces miR-351, miR-431, and miR-488 expressions, all of which exhibit specific RNA sequences similar to that of the human HCV genome, and is consequently able to functionally inhibit HCV replication [19]. miRNAs may also be downregulated by IFNs such as miR-21 [20]. miR-4661 directly affects the expression of IFN*α*, resulting in host inability to mount an appropriate antiviral innate immune response [21]. In psoriasis, IFN*γ*-mediated miR-149 inhibition leads to amplification of the skin inflammatory response to tumor necrosis factor-like weak inducer of apoptosis [22]. The IFN expression may be either suppressed or augmented by miRNAs. miRNAs also hold the power to alter IFN ability to regulate viral infections. The converse is also true, as seen by type I IFN-mediated inhibition of Dicer protein production at the post-transcriptional level, along with IFN*γ* induction of the Dicer expression [23]. Mice with Dicer knockout gene mutations demonstrate the abnormal IFN expression and are more susceptible to viral infections [24].

Interestingly, some lncRNA may function to inhibit or induce the IFN response. Viruses and bacteria pathogens have been shown to be able to counteract both positive and negative regulatory roles of lncRNA in the innate immune system. Therefore, cellular lncRNA regulation is able to directly modulate the IFN response. Some lncRNAs are able to influence IFN transcription and are implicated in IFN signaling. IFN*γ* transcription is strongly dependent on the lncRNA NeST expression, which is located in the proximity to the IFN*γ* gene [25, 26]. LncRNA IFNG-AS1 enhanced the expression of IFN*γ* in the immune response of brucellosis patients [27]. Mineo et al. reported that lncRNA INCR1 regulated IFN*γ* signal transduction across various tumors [28]. IFN-stimulated lncRNA lnc-MxA could promote IAV replication and inhibit IFN*β* production through the formation of an RNA-DNA triplex [29]. LncRNA linc00513 is identified a novel positive regulator of the type I IFN pathway by promoting STAT1 and STAT2 phosphorylation [30]. LncRNA RP11-2B6.2 was identified as a novel positive regulator of the type I IFN pathway and works through epigenetic inhibition of SOCS1 to suppress type I IFN pathway activation in lupus nephritis (LN) [31]. It has been reported that several lncRNAs can modulate transcription of proteins involved in IFN synthesis and ISGs. LncRNA-155 inhibited the PTP1B expression resulting in the upregulation of IFN*β* and several other ISGs (MxA, IFIT1, ISG15, IFI27, OAS3), thus contributing to host innate immunity during IVA infection [32]. LncRNA lncLrrc55-AS has been identified as an IFN-induced lncRNA, which promotes the production of type I IFN by increasing the IRF3 activity [33]. The lncRNA Malat1 was identified as gene that was downregulated by viral infection, which in turn promoted IRF3-triggered type I IFN production [34]. Some lncRNAs were found to negatively regulate the transcription of ISGs and promote viral replication. The overexpression of lnc641 inhibited IFN*α* expressions and reduced the expressions of phosphorylated JAK and STAT1 while also enhancing the replication of the pseudorabies virus [35]. LncRNA CMPK2 was identified as an ISG which negatively regulates the transcription of IFN-stimulated antiviral genes [36]. NRAV was recognized as lncRNA that is suppressed by the influenza virus, which decreased levels of NRAV leading to an increase in ISGs likely through its role in regulating chromatin remodeling factors [37]. Some lncRNAs can positively regulate ISG transcription and inhibit viral replication. LncRNA GAS5 was identified as an IFN-stimulated gene (ISG) and positively regulated IFN responses [38]. LncRNA Malat1, which contributes to the transcription of OSA2, OAS3, and OASL, was also found to control the IFN*α* response in SLE [39]. The novel lncRNA IVRPIE was identified a critical regulator of the host antiviral response through its impact of IFN*β*1 and ISGs [40]. Several lncRNAs can regulate ISGs by targeting peculiar transcriptional regulators. During infectious bursal disease virus (IBDV) infection, lncRNA loc107051710 promoted the production of IFN*α* and IFN*β* through IRF8 regulation, thereby promoting the antiviral activity of ISGs [41]. LncRNA lncITM2C is induced by HCV infection and promotes viral replication by contributing to the GPR55 expression [42].

Noncoding RNAs (ncRNAs) such as lncRNAs and miRNAs are central to transcriptome processing and are inherently implicated in numerous biological activities, including the pathophysiology of viral infections. There exists several interesting and complex crossregulation between lncRNAs and miRNAs, such as the presence of crosstalk between competitive endogenous RNAs (ceRNAs) which is mediated by shared common microRNA response elements (MRE). The current study seeks to attain deeper insights into the complex network between these molecules in the context of IFN overexpressing porcine alveolar macrophages.

## 2. Materials and Methods

### 2.1. DNA Construction, Cell Culture, and Transfection

Fragments containing the complete CDS of IFNA (ENSSSCT00000087954.1) or IFNB (ENSSSCT00045019653.1) or IFNG (ENSSSCT00000055560.2) were inserted into the expression vector pcDNA3.1 3D4/21 cells. Firstly, cell cultures were performed on 6-well plates (1.2 × 10^5^ cells per well) which each contained 10% fetal bovine serum-supplemented RPMI 1640 media (Gibco) in a humidified incubator in 5% CO2 at 37°C. Upon reaching the logarithmic growth stage (80-90% cell density), Lipofectamine®2000 was used to perform cell transfection. Cells were harvested after 24 h of transfection.

### 2.2. RNA and lncRNA Sequencing and Analysis

The TRIzol Reagent (Invitrogen, USA) was used to perform total RNA extraction in compliance to protocols stipulated by the manufacturer. Quantitative and qualitative analyses were then carried out on a total of 12 RNA samples. The RNA integrity number (RIN) of all the samples was between 9.8 and 10. An RNA library was prepared using 3 *μ*g of each RNA sample which was sequenced with the Illumina Hiseq 4000 platform prior to generating a paired-end read of 150 bp. After the sequencing was completed, the Novogene Corporation carried out the following preliminary analysis and operations: quality control analysis, reference genome reading, assembly of the transcriptome, analysis of coding potential, conservation analysis, prediction of target genes, quantification of gene expression levels, differential expression analysis and Gene Ontology (GO), and Enrichment analysis of Kyoto Genome and Genome Encyclopedia (KEGG).

All raw reads obtained after sequencing were stored in the FASTQ format. Clean reads were produced through removal of adapter sequences, low-quality reads, and reads possessing an unknown nucleotide “N.” Estimates of the GC, Q30, and Q20 content of cleaned data were performed to produce high-quality clean data for all subsequent analyses. The Tophat (v2.0.9) program was used to map all reads onto the porcine genome sequence assembly, with the Scripture (beta2) and Cufflinks programs used to assemble mapped reads for each sample via a reference-based approach.

The following lncRNA characteristics were predetermined prior to selection: (a) transcript length ≥ 200 nt, number of exons ≥ 2, and an open reading frame (ORF) length ≤ 300 nt; (b) transcript with paired end reads per kilobase per million mapping reads (FPKM) ≥ 0.5 (as determined via Cufflinks); (c) the selected transcripts were blasted against known pig lncRNAs in NONCODE and were completely mapped to the NONCODE and subsequently labeled as known lncRNAs; and (d) Coding Potential Calculator (CPC) [43], PFAM [44], and Coding Noncoding Index (CNCI) [45] were used to identify the coding and noncoding transcripts in this study.

Selected lncRNAs were grouped into three cohorts: (1) intergenic overlapping lncRNAs (lincRNAs) comprised of nongene overlapping lncRNAs (RefSeq or Ensembl), (2) intronic overlapping lncRNAs (intronic lncRNAs) were lncRNAs that were completely located in either the antisense or senseintrons of any protein-coding gene, and (3) antisense lncRNAs (antisense lncRNAs) were lncRNAs that overlapped with any RefSeq transcription exons on the reverse chain.

*cis*-Target genes were gene transcripts located within 100 kb upstream or downstream of the lncRNA. LncRNAs and target gene pairs with absolute value of reservation coefficient are greater than 0.95 as analyzed by Pearson's coefficient prior to selection.

Cufflinks were used to estimate transcript abundances in FPKM or single end reads per kilobase per million mapping reads (RPKM) [46]. In all differential expression tests, a gene was considered significant if the *q* value was less than 0.05.

### 2.3. Small RNA Sequencing and Analysis

A small RNA library was generated using 3 *μ*g RNA from each sample, clustered and sequenced on the Illumina Hiseq 2000 platform in order to generate 50 bp single-end reads which were then converted to the fasta format. MiReep2 was used to analyze these datasets [47]. Ensembl provided the porcine genome (Sscrofa 11.3), and the miRBase database (version 22) provided the miRNA reference [48]. The Novogene Corporation subsequently performed the following analyses: quality control analysis, read mapping onto the reference sequence, miRNA alignment, removal of source label, prediction of novel miRNA, small RNA annotation summaries, analyses of miRNA editing, miRNA quantification, miRNA family analysis, prediction of target genes, analysis of mRNA differential gene expression, and GO and KEGG enrichment analysis. The miRNA expression level was normalized using the following equation: TPM (transcripts per million reads) = read count × 1, 000, 000/total clean reads count. *q* ≤ 0.05 was set as the threshold for identification of differentially expressed (DE) miRNA.

### 2.4. Gene Ontology and Pathway Analyses

The GOseq *R* package was used to carry out the Gene Ontology (GO) enrichment analysis of differentially expressed genes (DEG) or lncRNA/miRNA target gene candidates, correcting for gene length bias [49]. Significant DEGs were identified if the corrected *p* value of GO terms was less than 0.05. KEGG is a database resource for processing complex biological system functions and utilities [50]. Statistically enriched DEGs or lncRNA/miRNA target gene candidates in KEGG pathways were analyzed with the KOBAS software [51].

### 2.5. Analyses of the lncRNA-miRNA-mRNA Network

**Figure 1:**
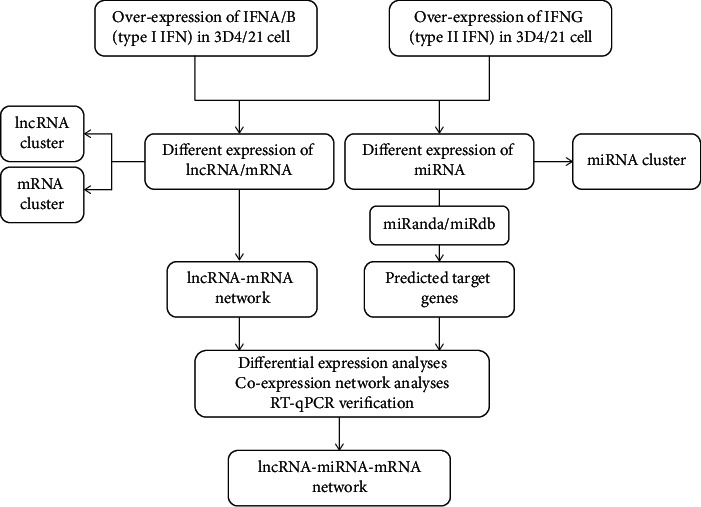
Workflow of lncRNA-miRNA-mRNA network analysis.

The lncRNA-miRNA-mRNA integrative analysis was performed according to Figure 1. The principle of miRNA interference or inhibition of target genes was used to predict miRNA-targeted lncRNA and miRNA-targeted mRNA. The correlation coefficients between miRNA and lncRNA as well as between miRNA and mRNA were calculated, and the negative correlations were selected. Based on the ceRNA research results, mRNA and lncRNA that were coregulated by the same miRNA were selected. Cytoscape 3.8.0 software was used to visualize the screening of lncRNA-miRNA-mRNA pairs.

### 2.6. RT-qPCR Validation of Differentially Expressed mRNAs, miRNAs, and lncRNAs

To verify the accuracy of high-throughput sequencing data, a total of 18 randomly selected genes which included eight lncRNAs, five miRNAs, and five mRNAs were chosen to undergo RT-qPCR verification. Similar methods of total RNA extraction as mentioned previously were performed. RNase-free DNase I (Fermentas) was first used to treat the extracted RNA prior to cDNA construction using random primers recommended by the RevertAid First Strand cDNA Synthesis Kit (Thermo Scientific) for the mRNA and lncRNA expression study. For the miRNA expression study, stem-loop RT primers were formulated in accordance to sRNA PrimerDB [52] and were subsequently used for reverse transcription. The standard SYBR Green PCR kit (Toyobo, Japan) was used to carry out qPCR assays on an ABI 7500fast (USA) system. The PCR conditions were 2 min at 95°C followed by 45 cycles of 30s at 95°C and 34 s at 60°C. Melting curves were obtained by increasing the temperature from 60°C to 95°C at 0.5°C for 15 s. LncRNAs and mRNA relative expressions were normalized against the GADPH gene while miRNA expressions were normalized against the U6 gene. An optimized comparative Ct (△△Ct) value method was used to determine the relative expression levels. Table S1 depicts all primers utilized for qPCR.

### 2.7. Statistical Analysis

All data is depicted in terms of means ± standard error of the mean (S.E.M.). Intergroup significances were calculated with either the *t*-test or analysis of variance (ANOVA). A *p* value of less than 0.05 was considered to be statistically significant, while a *p* value of less than 0.01 was determined to be extremely significant.

## 3. Results

### 3.1. Identification of lncRNAs in 3D4/21 Porcine Alveolar Macrophages

**Figure 2:**
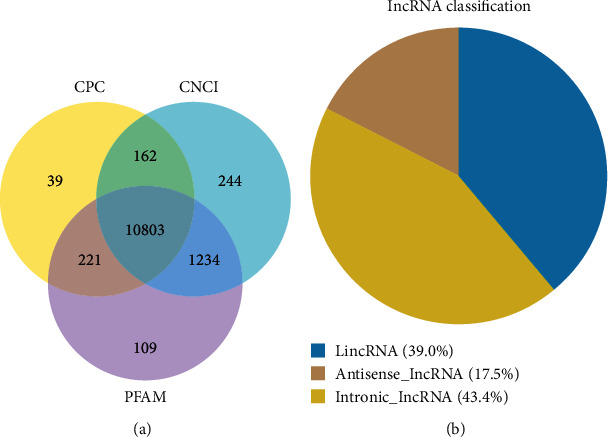
Identification and classification of lncRNAs IFN overexpressing 3D4/21 cells. (a) Venn diagrams were constructed for coding potential analysis based on strict standards. The lncRNA coding potential was analyzed using three tools (CPC, CNCI, and PFAM). Those shared by the three analysis tools at the same time were designated as candidate lncRNAs for subsequent analysis. (b) Classification of lncRNAs in the 3D4/21 cell. The lncRNAs identified in this study comprised of 39% large intergenomic noncoding RNA (lincRNA), 17.5% antisense lncRNA, and 43.4% intronic lncRNA.

**Figure 3:**
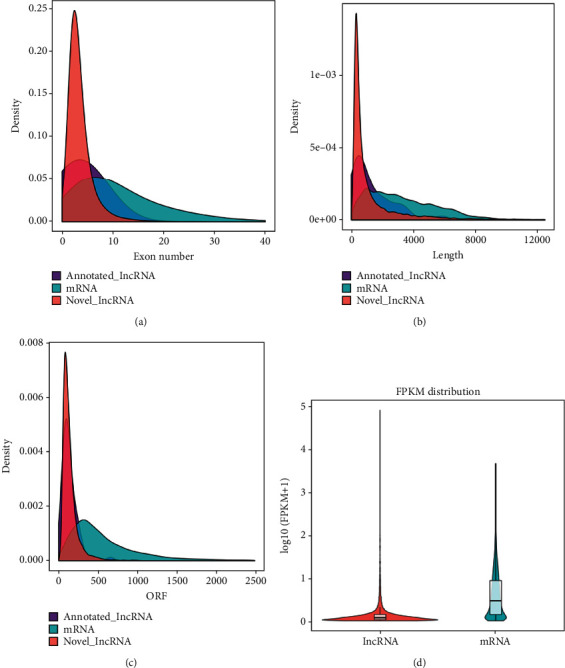
Comparison of the lncRNA and mRNA expression and genomic structure. (a) Exon distribution between lncRNA and mRNA in 3D4/21 cells. (b) Transcriptome length between lncRNA and mRNA in 3D4/21 cells. (c) The open reading frame (ORFs) length distribution in lncRNA and mRNA. (d) The lncRNA and mRNA expression levels as described in terms of log10 (FPKM+1).

10803 lncRNAs from 3D4/21 cells were determined postassembly (Figure 2(a)). Including 63 lncRNAs mapped onto NONCODE, a total of 10866 lncRNAs were identified. The 10866 lncRNAs comprised of 39% large intergenic noncoding RNAs (lincRNAs), 17.5% intronic lncRNAs, and 43.3% antisense lncRNAs (Figure 2(b)). A detailed comparison between lncRNAs and mRNAs was done with focus on their expression levels, sequence conservation, and genomic structure differences. We discovered that both novel and annotated lncRNAs possessed between two to three exons which were distributed along the length of the transcript (Figure 3(a)). Most of the lncRNA transcript lengths were distributed along an area of shorter length in contrast to that of the mRNAs (Figure 3(b)). A majority of the lncRNAs possessed shorter ORF in contrast to those of mRNAs and showed a lower expression level (Figures 3(c) and 3(d)).

### 3.2. Differentially Expressed lncRNAs in 3D4/21 Cells with Overexpressed Type I and II IFN

**Figure 4:**
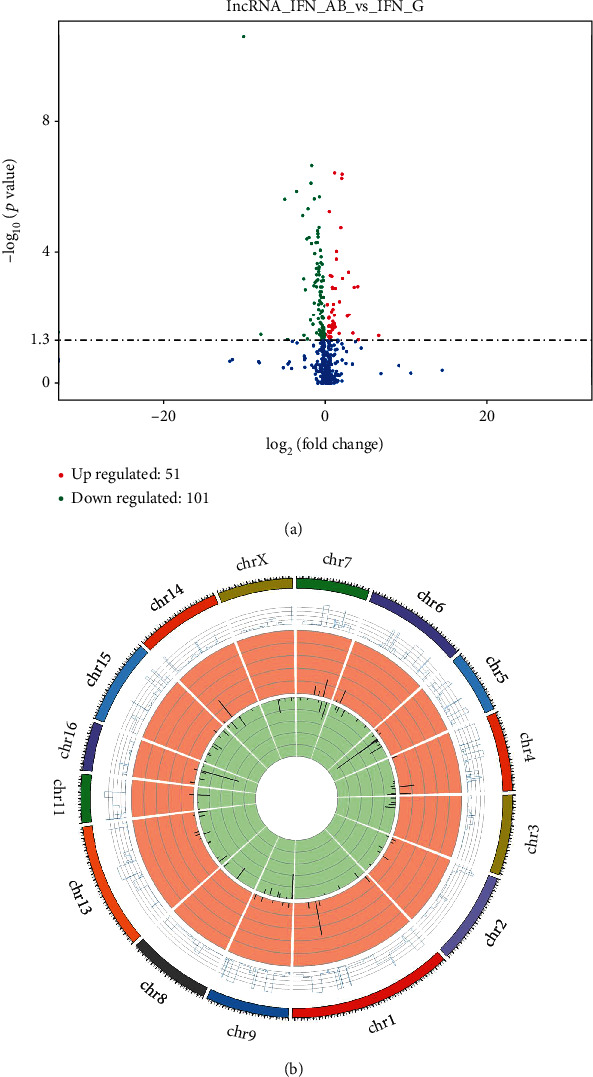
Differential lncRNA expression between type I and type II IFN. (a) Volcano plot of differentially expressed lncRNAs between type I and type II IFN overexpression in 3D4/21 cells. Red and green circles denoted markedly upregulated and downregulated lncRNAs, respectively. (b) The distribution of differentially expressed lncRNA on chromosomes. The Circos diagram showed the chromosome distribution of the differential transcripts: the outermost ring is the chromosome; the second circle is the FPKM sample of the comparative combination on the chromosome; the third circle shows the distribution of significantly upregulated chromosomal transcripts; the fourth circle shows the distribution of significantly downregulated chromosomal transcripts.

We identified 152 differentially expressed (DE) lncRNAs between 3D4/21 cells which overexpressed type I IFN and type II IFN (*q* value <0.05). Among the 152 lncRNAs, 51 were upregulated (more highly expressed in the type I IFN group), and 101 were downregulated (more highly expressed in the type II IFN group) (Figure 4(a)). We also mapped the readings to each chromosome, with the results corresponding to chromosome length (Figure 4(b)). Additionally, we identified 18 upregulated DE lncRNAs and 27 downregulated DE lncRNAs between type I IFN and type II IFN (∣log2 fold change | ≥1, *q* value <0.05). The fold change in the expression of the DE lncRNAs ranged between -10.1521 and 4.0219 (Table 1).

### 3.3. GO Enrichment Analysis for Differentially Expressed lncRNAs

**Figure 5:**
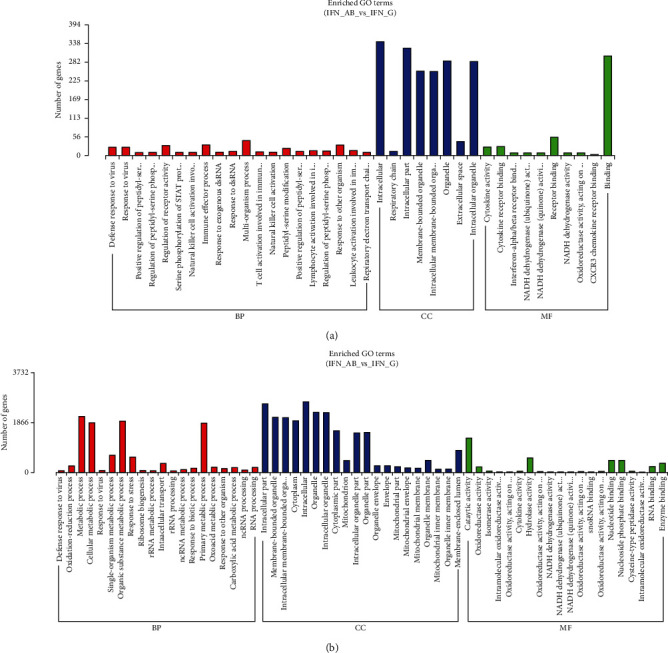
GO enrichment results of target genes of lncRNAs. The categories of biological processes, cellular components, and molecular functions of the target genes of differentially expressed lncRNAs and mRNAs ((a), cis; (b), trans). BP: biological processes; CC: cellular component; MF: molecular function.

The GO classification of downregulated and upregulated genes was contrasted using GOseq (corrected *p* value <0.05) (Young et al., 2010). 152 DE lncRNAs and coexpressed mRNAs possessed enriched GO terms of 4780 in all of the lncRNA *cis*-targets. Among them, 3559 of the enriched GO terms were categorized as biological process (BP), 486 of the enriched GO terms were categorized as cellular component (CC), and 735 of the enriched GO terms were categorized as molecular function (MF). The top 20 significantly enriched GO terms of BP, 8 terms of CC, and 10 terms of MF (corrected *p* value <0.05) are shown in Figure 5(a). Viral defense response represented the most significantly enriched GO term between type I IFN and type II IFN groups. 11722 GO terms were noted to be enriched for *trans*-targets of 152 DE lncRNAs and coexpressed mRNAs. Among them, 8502 of the enriched GO terms were categorized as BP, 1055 of the enriched GO terms were categorized as CC, and 2165 of the enriched GO terms were categorized as MF.  Figure 5(b) depicts the respective top 20 significantly enriched GO terms of BP, CC, and MF. The top three GO terms were intracellular part, membrane-bounded organelle, and intracellular membrane-bounded organelle.

### 3.4. KEGG Pathway Enrichment Analysis for Differentially Expressed lncRNAs

**Figure 6:**
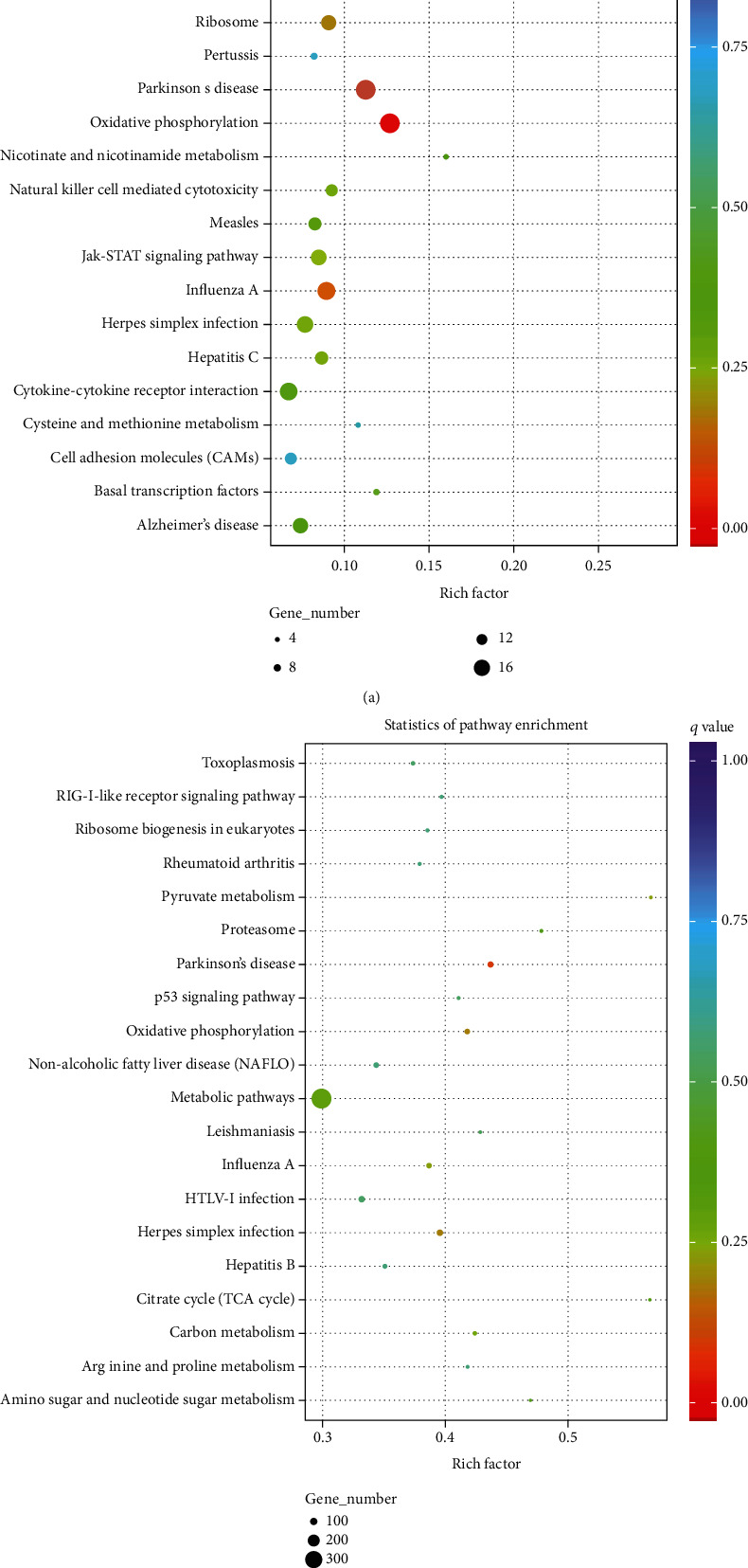
KEGG enrichment for target genes of lncRNAs. Scatter plot of the top 20 pathways enriched for differentially expressed lncRNAs and mRNA in 3D4/21 cells ((a), cis; (b), trans). Richness factor is depicted via the abscissa, while enrichment pathway items are represented via the ordinate. The *q* value represents the corrected *p* value.

**Figure 7:**
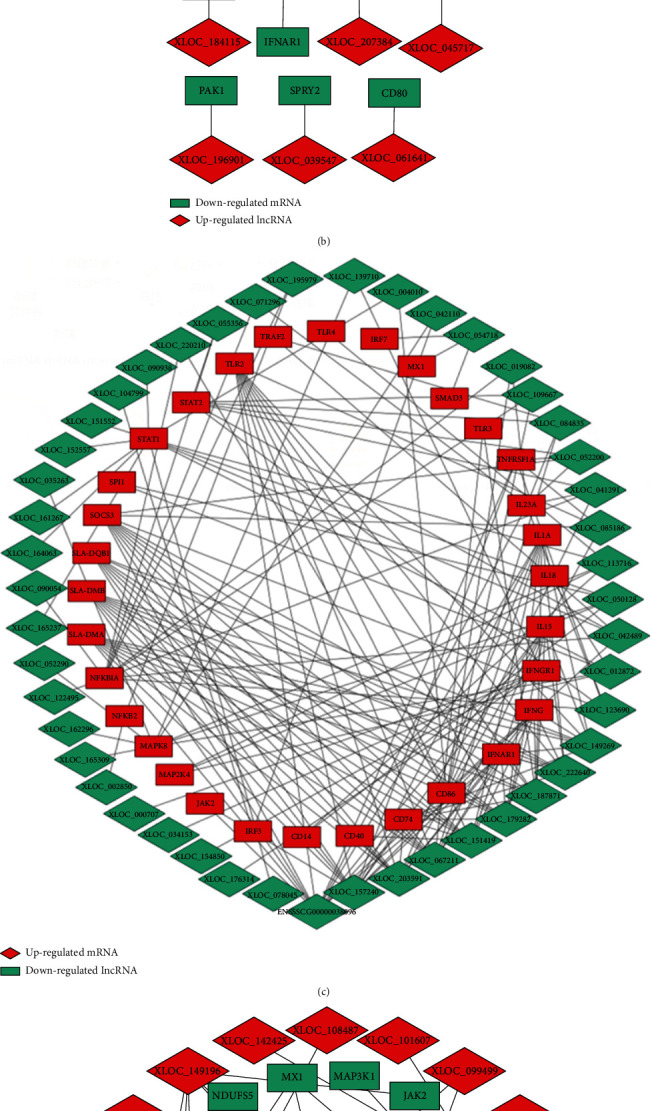
The interaction network analysis of DE lncRNA and DE target immune-related genes. (a, b) DE lncRNA and DE *cis*-target immune-related genes interaction network. (c, d) DE lncRNA and DE *trans*-target immune-related genes interaction network.

The KEGG pathway analysis was used to enrich differentially coexpressed lncRNAs and target mRNAs in the type I IFN and type II IFN groups to determine the relevant biological pathways. The pathways of the DE lncRNA target genes were significantly enriched for the Toll-like receptor signaling pathway, Jak-STAT signaling pathway, cytokine-cytokine receptor interaction, and cell adhesion molecules in the *cis*-targets of lncRNAs and target mRNAs (Figure 6(a)). For the *trans*-targets of lncRNAs and target mRNAs, Parkinson's disease, herpes simplex infection, HTLV-I infection, Rheumatoid arthritis, nonalcoholic fatty liver disease, p53 signaling pathway, RIG-I-like receptor signaling pathway，and nonalcoholic fatty liver disease were identified (Figure 6(b)). The interaction networks between DE lncRNA and DE target immune-related genes were constructed using Cytoscape (Figure 7). We uncovered that several genes were involved in the Toll-like receptor signaling pathway, Jak-STAT signaling pathway, natural killer cell mediated cytotoxicity, influenza A, and p53 signaling pathway. These genes included genes such as CD80, CD82, CXCL11, CXCL10, CXCL8, c-fos, JAK1, ICAM1, STAT2, TLR3, IRF7, IRF3, MX1, SLA-DQA/DQB/DRB, IL8, IL18, MAP 2 K4, NF-KB1A, and TP53I3 (Table S2). Some of these pathways may be associated with physiological antiviral processes.

### 3.5. Differentially Expressed miRNAs in 3D4/21 Cells with Overexpressed Types I and II IFN

**Figure 8:**
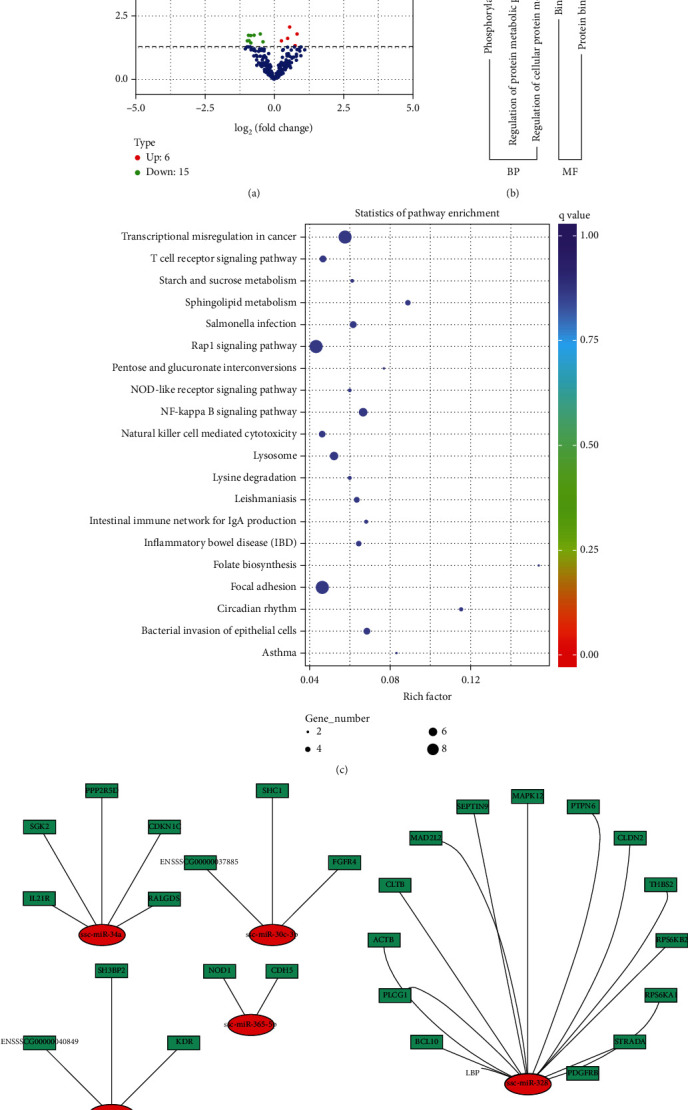
Differential miRNA expression between type I and type II IFN. (A) Volcano plot of differentially expressed miRNAs between type I and type II IFN overexpression in 3D4/21 cells. Red and green circles depict markedly upregulated and downregulated miRNAs, respectively. (b) DE miRNA-targets are enriched in the GO terms. (c) DE miRNA-targets enriched in the KEGG pathway. (d) DE lncRNA and their target immune-related genes interaction network.

We identified 21 differentially expressed (DE) miRNAs between type I IFN and type II IFN (*q* value <0.05). Among these 21 miRNAs, 6 were expressed at a higher degree in the type I IFN group, and 15 possessed elevated expressions in the type II IFN group (Figure 8(a)). Furthermore, we identified 1 DE miRNA (ssc-miR-221-5p) which was raised in the type I IFN group and 5 DE miRNAs (ssc-miR-885-5p/3p, ssc-miR-29a/29c, ssc-miR-491, and ssc-miR-708-3p) which were raised in the type II IFN group (∣log2 fold change | ≥1, *q* value <0.05). The DE miRNA fold change expressions ranged between -1.7336 and 1.7614 (Table 2).

### 3.6. GO Enrichment Analysis for Differentially Expressed miRNAs

21 DE miRNAs and coexpressed mRNAs possessed 4686 enriched GO terms across all target genes. Among them, 3428 of the enriched GO terms were classified to represent BP, 497 of the enriched GO terms were recognized as CC, and 761 of these represented MF.  Figure 8(b) demonstrates the top two significantly enriched GO terms of MF and three terms of BP (corrected *p* value <0.05). Binding and protein binding were the most significantly enriched GO term between type I IFN and type II IFN groups.

### 3.7. KEGG Pathway Enrichment Analysis for Differentially Expressed miRNAs

The KEGG pathway analysis was performed to uncover the relevant biological pathways of the enriched differentially coexpressed miRNAs and target mRNAs across type I IFN and type II IFN groups. The pathways of the DE miRNA target genes were significantly enriched for the NOD-like receptor signaling pathway, T cell receptor signaling pathway, Rap1 signaling pathway, NF-kappa B signaling pathway, bacterial invasion of epithelial cells, and natural killer cell-mediated cytotoxicity (Figure 8(c)). In addition, we used Cytoscape to demonstrate immune-related DEG targeted by DE miRNA (Figure 8(d)). We uncovered that several genes were involved in the NF-kappa B signaling pathway, bacterial invasion of epithelial cells, Salmonella infection, inflammatory bowel disease, Rap1 signaling pathway, intestinal immune network for IgA production, T cell receptor signaling pathway, natural killer cell-mediated cytotoxicity, and NOD-like receptor signaling pathway components such as BCL10, LBP, PLCG1, ACTB, CLTB, MAPK12, FGFR4, IL21R, PLCG1, ITGB7, and NOD1 (Table S3). Some of these pathways may be associated physiological antiviral and antibacterial response mechanisms.

### 3.8. Interaction Network among lncRNAs, miRNAs, and mRNAs

**Figure 9:**
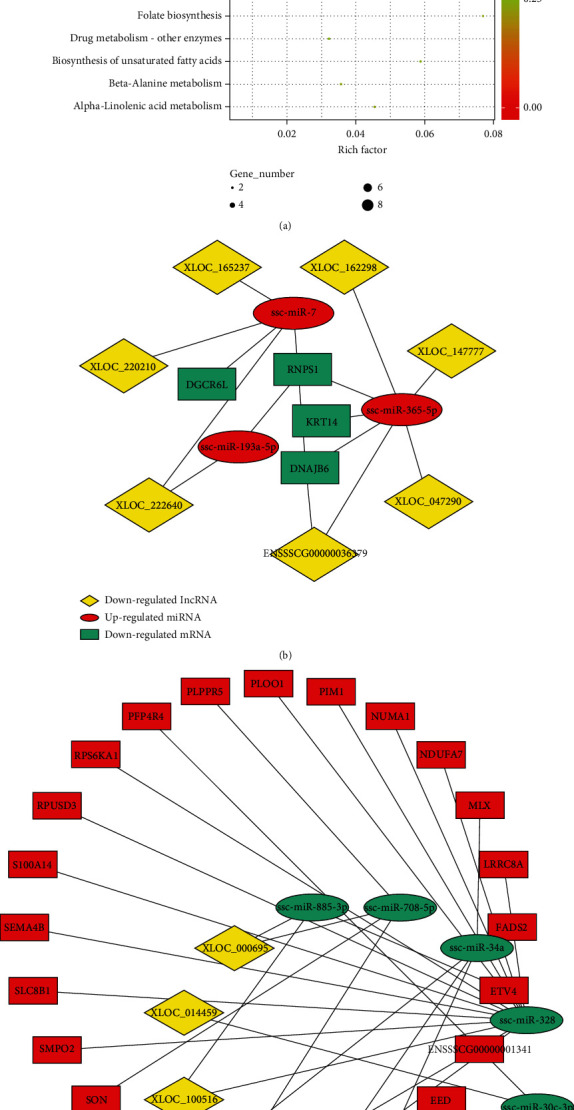
The interaction network analysis of lncRNA-miRNA-mRNA. (a) The DEGs in the interaction pairs of lncRNA-miRNA-mRNA enriched in the KEGG pathway. (b, c) DE lncRNA, miRNA, and DE cis/trans target immune-related genes interaction network ((b), miRNA (up-regulated); (c), miRNA (downregulated)).

In order to further explore the interaction among DE lncRNAs, DE miRNAs, and DEGs, we constructed putative endogenous competitive RNA (ceRNA) by incorporating lncRNA-miRNA-gene expressions in porcine alveolar macrophages with overexpressed IFN levels. A total of 7 lncRNAs, 3 miRNAs, and 4 mRNAs were involved in 17 lncRNA (downregulated)–miRNAs (upregulated)–gene (downregulated) pairs in porcine alveolar macrophages overexpressing IFN (Table 3). A total of 6 lncRNAs, 7 miRNAs, and 24mRNAs were involved in 29 lncRNA (upregulated)–miRNAs (downregulated)–gene (upregulated) pairs in porcine alveolar macrophages overexpressing IFN (Table 3). KEGG pathway analysis demonstrated that these DEGs in the ceRNA network were associated with diseases such as nonalcoholic fatty liver disease, Huntington's disease, Parkinson's disease, and Alzheimer's disease (Figure 9(a)). The ceRNA network of the NOD-like receptor signaling pathway, mTOR signaling pathway, PPAR signaling pathway, Jak-STAT signaling pathway, and MAPK signaling pathway including MIX, CLTB, NDUFA7, RPS6KA1, PIM1, and FADS2 genes were imported to Cytoscape for visualization (Figures 9(b) and 9(c)).

Additionally, lncRNAs including XLOC_211306, XLOC_100516, XLOC_00695, XLOC_149196, and XLOC_014459 were expressed at a higher degree in the type I IFN group, while XLOC_222640, XLOC_047290, XLOC_147777, XLOC_162298, XLOC_220210, and XLOC_165237 were expressed at a higher degree in the type II IFN group. These lncRNAs were found to act as “sponges” for some miRNAs, such as miR-34a, miR-328, miR-885-3p, miR-149, miR-30c-3p, miR-30b-5p, miR-708-5p; miR-193a-5p, miR-365-5p, and miR-7, in addition to targets FADS2, RPS6KA1, PIM1, and NOD1. They were found to be associated with several immune-related signaling pathways, such as the PPAR signaling pathway, mTOR signaling pathway, Jak-STAT signaling pathway, and the NOD-like receptor signaling pathway (Table 3, Table S4). These results indicate that porcine alveolar macrophages overexpressing IFN were affected by host immune-related factors, including lncRNAs, miRNAs, and mRNAs via the ceRNA network.

### 3.9. Verification of lncRNA, miRNA, and mRNA Expression Profiles Using RT-qPCR

**Figure 10:**
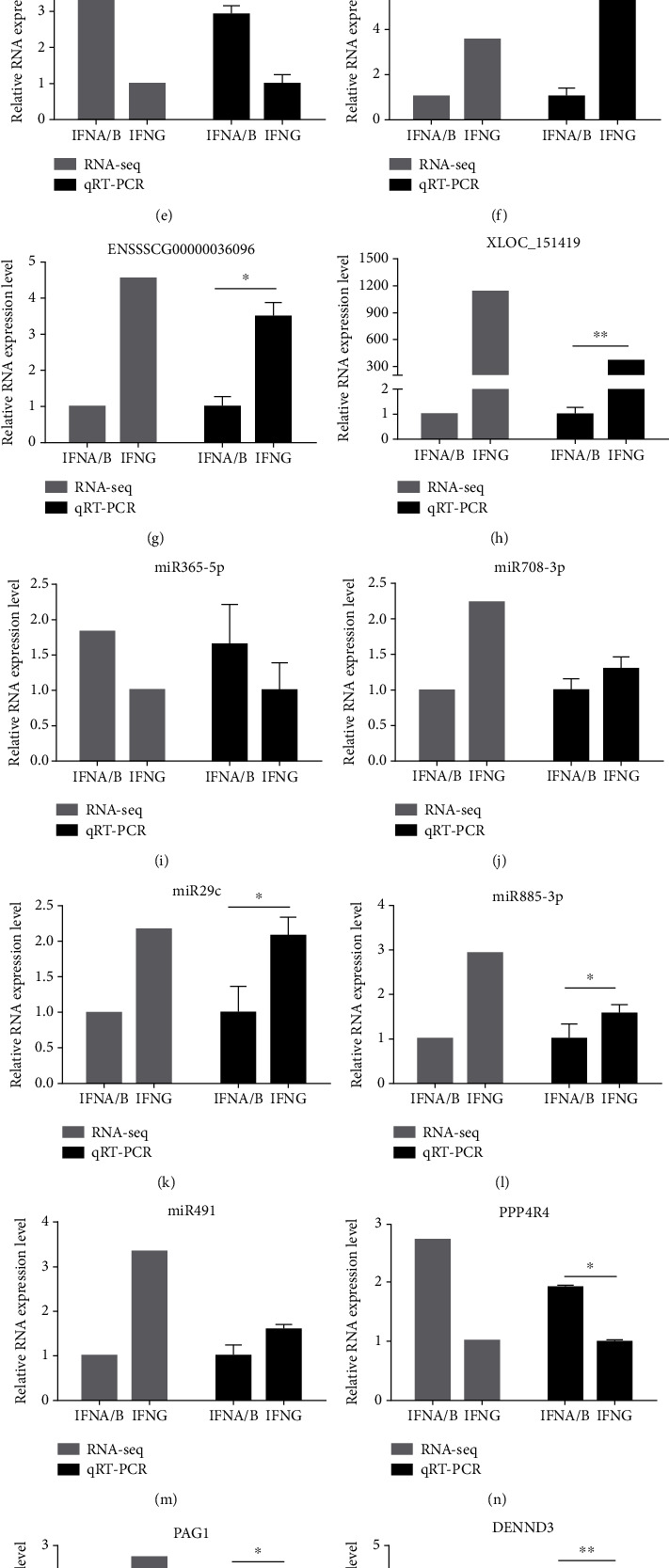
Validation of differentially expressed lncRNAs, miRNAs, and mRNAs by RT-qPCR. RT-qPCR results of select lncRNAs (a)–(h), miRNAs (i)–(m), and mRNAs (n)–(r).

The accuracy of the lncRNA-seq and miRNA-seq results were verified through RT-qPCR evaluation of 8 candidate lncRNAs, 5 candidate miRNAs, and 5 candidate mRNAs which were randomly selected from DEGs across type I IFN and type II IFN groups. Table S1 depicts all primers used. The results of RT-qPCR were identical to those of RNA-seq. The values of fold change representative lncRNAs, miRNAs, and mRNAs in RT-qPCR similarly trends as the log2 (foldchange) in RNA-seq (Figure 10).

## 4. Discussion

IFNs are potent cytokines with broad-spectrum antiviral effects. CeRNAs are critical in the IFN function and are indispensable mediators of the innate immune antiviral response. Our study analyzed lncRNA, miRNA, and mRNA expression patterns in IFN-overexpressing 3D4/21 cells using high-throughput RNA-seq. We uncovered 152 differentially expressed (DE) lncRNAs and 21 DE miRNAs between type I and type II IFN overexpressing porcine alveolar macrophages and further identified 36 DE lncRNAs, 12 DE miRNAs, and 72 mRNAs which were involved in the DE lncRNA-miRNA-mRNA interaction networks. Several representative DE lncRNAs, miRNAs, and mRNAs selected for RT-qPCR verification demonstrated similar trends as noted by the RNA-seq data analysis. Additionally, the most significantly altered GO categories and KEGG pathways were identified in order to construct DE lncRNA-miRNA-mRNA interaction networks that were able to predict ceRNA function.

CeRNA functions in relation to the IFN response is cell-dependent and involves a cascade of interactions that regulate IFN production and signal response activity. It is well known that IFN binding to IFN receptors induces an immune response via the Jak-STAT signaling pathway. However, other signaling pathways such as MAPK, mTOR, and PKC which are activated by other receptors have also been implicated in IFN activity [53]. Functional annotation of the DE lncRNA and miRNA target genes indicates the involvement of the inflammatory response and immune related pathways. DE lncRNA target genes were significantly enriched in pathways such as the Toll-like receptor and the Jak-STAT signaling pathways. The p53 signaling pathway and RIG-I-like receptor signaling pathway were significantly enriched for *trans*-targets of lncRNAs and target mRNAs. For miRNAs and target mRNAs, the Rap1 signaling pathway, NF-kappa B signaling pathway, T cell receptor signaling pathway, and NOD-like receptor signaling pathway were identified. For lncRNA and miRNA target genes, the NOD-like receptor signaling pathway, mTOR signaling pathway, MAPK signaling pathway, PPAR signaling pathway, and the Jak-STAT signaling pathway were identified.

The interaction between IFN and noncoding RNA gene expressions has been demonstrated to be vital in the ability to mount an antiviral innate immune response [54–56]. CeRNA research is known to be riddled with complexities especially with regards to lncRNA as the functions of most lncRNAs remain unknown. There is no existing lncRNA database that allows for accurate annotation. Some lncRNAs are known to regulate the expressions of mRNA located in their proximity, and their expressions were found to be highly correlated to these mRNAs [57]. Based on this knowledge, we searched for target lncRNA genes to predict their functions.

Inflammation and immune regulation involve complex regulatory networks. This investigation constructed coexpression networks of lncRNAs and the encoding gene transcripts in order to predict the likely biological features of DE lncRNAs. DE lncRNAs and mRNAs were enriched in some immune-associated signaling pathways, such as the Toll-like receptor signaling pathway, Jak-STAT signaling pathway, natural killer cell mediated cytotoxicity, and the p53 signaling pathway. Therefore, several roles of many target genes have been studied primarily in relation to the immune system, such as CD80, CD82, CXCL11, CXCL10, CXCL8, c-fos, JAK1, ICAM1, STAT2, TLR3, IRF7, IRF3, MX1, SLA-DQA/DQB/DRB, IL8, IL18, MAP 2 K4, NF-KB1A, and TP53I3. Antigen presenting cells (APC) such as macrophages, dendritic cells, B cells, and T cells are all known to express costimulatory CD80 molecules. The herpes simplex virus type 1 (HSV-1) has been found to escape immune detection through ICP22-mediated CD80 inhibition [58]. CD82 was identified as to be a useful indicator of poor prognosis in breast cancer patients [59]. IFN-*γ* is known to induce a multitude of genes while itself being induced by TNF*α* [60]. TNF*α* can enhance IFN-*γ*-induced class II MHC expression, such as HLA-DRA [61]. IFN-*γ* has been reported to upregulate CD74, a class II MHC molecule that is highly expressed in melanoma tissues [62, 63]. IFN-induced CXC chemokines, such as CXCL9, CXCL10, and CXCL11, are multifunctional chemokines. CXCL10, also known as the IFN-*γ* inducible protein 10 (IP-10), is a chemokine involved in the immune response that directs T cells to the site of inflammation [64]. CXCL10 can also be induced by IFN-*γ* in monocytes, lymphocytes, keratinocytes, and endothelial cells [65].

FADS2 (fatty acid desaturase 2) is involved in catalyzing the biosynthesis of highly unsaturated fatty acids [66]. IFN signaling can inhibit sterol synthesis in macrophages [67]. It was found that RPS6KA1 (Msk1) was phosphorylated on Serine 376 in an IFN*γ*-dependent manner, and the amount of RPS6KA1 protein previously detected did not change after IFN*α* stimulation [68]. Nucleotide-binding oligomerization domain 1 (NOD1) is an intracellular sensor of peptides derived from the peptidoglycan component of the bacterial cell wall. NOD1 activates type I IFN signaling to mediate mucosal host defense against Helicobacter pylori infection [69]. NOD1 regulates IFN*γ* production to deter the development of colitis-related tumors [70]. The expression of the PIM1 gene could be upregulated upon T lymphocytes exposure to IFN*α* [71]. IFN*γ* could stimulate expression of PIM1 RNA and protein in human factor-dependent cell line MO7e [72]. In this study, PIM1, RPS6KA1, and FADS2 were targeted by miR-328, and PIM1was determined to be a direct target of miR-328 [73]. NOD1 was targeted by miR-365-5p.

Some immune-related miRNAs such as miR-7, miR-221-5p, miR-365-5p, miR-193a-5p, and let-7d-5p were more highly expressed in the type I IFN group. On the other hand, miR-885-5p/3p, miR-29a, miR-29c, miR-155-3p, miR-708-3p/5p, miR-491, miR-149, miR-328, miR-30c-3p, miR-30b-5p, miR-34a, miR-574, and miR-9843-3p were more highly expressed in the type II IFN group. It has been found that miR-221 was able to enhance IFN*α*'s anti-HCV effect through SOCS1 and SOCS3 targeting [74]. miR-29c appeared to be protective against HCV infection by its effect on enhancing the STAT3-mediated type I IFN response in Huh7 cells [75]. miR-29 suppresses the immune response to intracellular bacterial infection by targeting IFN-*γ* [76]. IFN-*γ* stimulate fibroblasts while significantly increasing the miR-7 expression [77]. IFN-*γ* is also a critical cytokine in PRRSV infection and vaccine response [78]. Porcine miR-29a and IFN-*γ* expressions in animals injected with the modified live PRRSV vaccine were elevated 3 days after inoculation [17]. These findings form the foundation of a more effective PRRSV vaccine.

Some lncRNAs act as miRNA “sponges” by inhibiting the interaction between these miRNAs and target genes through posttranscriptional regulation. LRRC8A (leucine rich repeat containing 8 VRAC subunit A) is a common target gene of XLOC_100516 and ssc-miR-328 and is involved in B cell and T cell development. LRRC8A-deficient mice were found to have a weak immune response against HSV-1 (herpes simplex virus 1) infections as well as an impaired IFN response [79]. LncRNA lnc-ISG20 acts as a ceRNA by binding to miR-326 and releasing ISG20 from miR-326-mediated downregulation [80]. In macrophages infected with *Listeria monocytogenes*, there was an upregulated miR-1 expression which resulted in downregulated lncRNA Sros1 and subsequently upregulated STAT1 protein. This resulted in an overall stronger IFN*γ* immune response against intracellular bacterial infection [81].

Cell survival is also altered by IFNs through its action on apoptosis, which has been demonstrated to be a result of STAT and IRF regulation of the type I IFN response [82, 83]. IRF-1 is a central mediator of IFN-*γ*-induced apoptosis [84, 85]. Sequencing data demonstrated IRF1 to be highly expressed in the IFN-*γ* group. The ability of IFN to promote apoptosis confers a significant protective effect against pathological infections. Among the common target genes of lncRNA and miRNA in the ceRNA network, some genes such as RNPS1 (RNA binding protein with serine-rich domain 1), DNAJB6 (DnaJ heat shock protein family (Hsp40) member B6), PIM1 (Pim-1 protooncogene, serine/threonine kinase), S100A14 (S100 calcium-binding protein A14), and NUMA1 (nuclear mitotic apparatus protein 1) were found to regulate apoptosis.

In conclusion, we identified genome-wide lncRNA and miRNA expression patterns in 3D4/21 cells overexpressing type I and type II IFN using high-throughput sequencing technology. We hypothesized that lncRNA and miRNA may possess critical functions roles in IFN-overexpressing porcine alveolar macrophages by targeting immune- and apoptosis-related genes through the ceRNA network. The integration analysis of lncRNA, miRNA, and mRNA in 3D4/21 cells overexpressing IFN provided insights into the molecular mechanisms underscoring the role of IFN in the physiological immune response. Nevertheless, further studies verifying the regulatory mechanisms of lncRNAs and miRNAs are necessary.

## Figures and Tables

**Table 1:** DE lncRNAs between type I IFN and type II IFN (∣log2 fold change | ≥1, *q* value <0.05).

lncRNA gene_id	IFN_AB_FPKM	IFN_G_FPKM	log2 (fold change)	*p* value	*q* value
XLOC_211602	43.36269267	2.669382333	4.021876484	0.001129464	0.005491452
XLOC_211597	21.8378445	1.820273333	3.584603455	0.0011786	0.005670808
XLOC_184115	149.2883267	14.16217267	3.397986845	0.031121919	0.084793931
XLOC_211602	32.46948567	4.324115	2.908607735	0.008410133	0.028455161
XLOC_140733	4.626857667	0.633625	2.868331555	0.000407351	0.002412352
XLOC_167604	1.66554	0.261060667	2.673532991	0.009486085	0.031417811
XLOC_045711	4.655611167	1.054105333	2.142951532	0.000631107	0.003425229
XLOC_149196	3.044239833	0.723581667	2.072854263	4.27*E*-07	2.16*E*-05
XLOC_211602	143.4954275	35.229205	2.026160942	5.69*E*-07	2.58*E*-05
XLOC_140789	60.609864	15.964264	1.924706563	1.83*E*-05	0.000240674
XLOC_182385	2.431886167	0.714496333	1.767077187	0.003266371	0.013108621
ENSSSCG00000032301	69.43411667	21.88469333	1.665722542	0.031984145	0.086681667
XLOC_211597	42.95031817	16.29083933	1.398607883	9.60*E*-05	0.000778055
XLOC_045717	110.4955812	42.34796133	1.383624252	0.000162899	0.001172263
XLOC_118537	4.0887745	1.712511	1.255555244	0.001272292	0.006037955
XLOC_006281	162.8222592	70.68881233	1.203744134	0.020304103	0.05972084
XLOC_101607	132.2356262	58.78432867	1.169607409	3.86*E*-07	2.02*E*-05
XLOC_044990	2.658949167	1.250159667	1.088743833	0.016602885	0.050389029
XLOC_026880	109.0063407	51.39015567	1.084848129	0.008884082	0.029737973
XLOC_142425	194.5797017	96.04973367	1.018507699	0.003829714	0.014907982
XLOC_151419	0.037438167	42.60000333	-10.15212804	2.89*E*-11	7.02*E*-08
XLOC_090366	0.010179333	2.601936333	-7.997798772	0.033904237	0.091073148
XLOC_203591	0.363516	12.03647667	-5.049250471	2.47*E*-06	6.61*E*-05
XLOC_156006	0.299248	7.695048667	-4.684516941	0.047489582	0.11914053
XLOC_179282	0.234744333	2.881586	-3.61770084	1.41*E*-06	4.75*E*-05
XLOC_157240	2.4332555	17.18107833	-2.819860866	7.65E-06	0.00013828
XLOC_187871	0.523616833	3.407369	-2.702074809	0.000657775	0.003545569
XLOC_214310	0.383863333	2.455601	-2.677411497	0.036087785	0.095692258
XLOC_039547	0.827454667	4.704943333	-2.507425168	0.001402749	0.006514006
XLOC_067211	0.775256833	3.874207	-2.321154797	4.01*E*-05	0.000409809
XLOC_184115	9.645355	45.96352333	-2.252583149	0.046083195	0.11648375
ENSSSCG00000036096	8.387311667	38.206422	-2.187534785	4.75*E*-06	9.96*E*-05
XLOC_085186	2.854869667	11.754609	-2.041729762	3.65*E*-05	0.000383711
XLOC_064393	0.698760667	2.536555333	-1.860000326	0.012493815	0.039560357
XLOC_047290	1.0963875	3.891838	-1.827693873	7.98*E*-07	3.31*E*-05
XLOC_071296	1.0572235	3.574042333	-1.757276324	5.56*E*-05	0.000519323
XLOC_149269	1.569842167	5.155544	-1.715505149	2.31*E*-07	1.45*E*-05
XLOC_151318	7.2517325	21.69476033	-1.580949035	0.01701197	0.051437935
XLOC_161309	1.091436667	3.096219333	-1.504279258	0.008623439	0.02905726
XLOC_200311	3.122596	8.660686667	-1.471735488	0.001059095	0.005218831
XLOC_082022	1.143892	3.080398667	-1.42916623	0.003540934	0.013979719
XLOC_123690	4.344915333	11.615328	-1.418629925	2.40*E*-06	6.48*E*-05
XLOC_052200	2.652597	6.758286667	-1.349252038	0.00011198	0.000873914
XLOC_084835	1.444592667	3.588942333	-1.31289599	0.000838627	0.004282989
XLOC_195979	6.011876333	13.92847	-1.212149553	5.18*E*-05	0.000492813
XLOC_002850	4.874692667	11.02583333	-1.177504529	0.000312666	0.001958251
XLOC_160076	1.4611615	3.230162	-1.144490875	0.001141319	0.005541073
XLOC_054718	1.605191167	3.547530667	-1.144070034	0.000487068	0.00276327
XLOC_019082	2.982730833	6.547019	-1.134204373	0.000742959	0.003889592
XLOC_092267	1.763106833	3.836783	-1.121777276	0.031067655	0.084669809
XLOC_198883	3.5318105	7.460047	-1.078776783	0.000225348	0.00151227
XLOC_122495	7.216612833	14.91292967	-1.047169943	5.16*E*-05	0.000491086
XLOC_054718	5.375565667	10.84738367	-1.012858632	2.29*E*-05	0.000278999

**Table 2:** DE miRNAs between type I IFN and type II IFN (∣log2 fold change | ≥1, *q* value <0.05).

sRNA	IFN_AB_readcount	IFN_G_readcount	log2 fold change	*p* val	*p* adj
ssc-miR-221-5p	23457.41747	6400.235784	1.7614	2.62*E*-15	5.97*E*-13
ssc-miR-885-5p	27.87665416	114.8575259	-1.7336	4.28*E*-07	4.88*E*-05
ssc-miR-491	52.25463333	174.1419016	-1.4895	8.25*E*-06	0.00026863
ssc-miR-885-3p	16.13439294	47.19168741	-1.4065	1.01*E*-06	5.76*E*-05
ssc-miR-29a	5975.857608	14666.54429	-1.2042	7.59*E*-07	5.76*E*-05
ssc-miR-708-3p	41.98103345	93.72264407	-1.0839	7.05*E*-06	0.00026801

**Table 3:** Gene list of pairs of lncRNA-miRNA-mRNA.

lncRNA- (downregulated-) miRNA- (upregulated-) gene (downregulated) pairs		
lncRNA gene_id	sRNA	gene_name
XLOC_222640	ssc-miR-193a-5p	RNPS1
XLOC_047290	Ssc-miR-365-5p	DNAJB6
XLOC_047290	ssc-miR-365-5p	KRT14
XLOC_047290	ssc-miR-365-5p	RNPS1
XLOC_147777	ssc-miR-365-5p	DNAJB6
XLOC_147777	ssc-miR-365-5p	KRT14
XLOC_147777	ssc-miR-365-5p	RNPS1
ENSSSCG00000036379	ssc-miR-365-5p	DNAJB6
ENSSSCG00000036379	ssc-miR-365-5p	KRT14
ENSSSCG00000036379	ssc-miR-365-5p	RNPS1
XLOC_162298	ssc-miR-365-5p	DNAJB6
XLOC_162298	ssc-miR-365-5p	KRT14
XLOC_162298	ssc-miR-365-5p	RNPS1
XLOC_220210	ssc-miR-7	DGCR6L
XLOC_165237	Ssc-miR-7	DGCR6L
ENSSSCG00000036379	Ssc-miR-7	DGCR6L
XLOC_222640	Ssc-miR-7	DGCR6L

lncRNA (upregulated)–miRNA (downregulated)–gene (upregulated) pairs		
XLOC_211306	ssc-miR-34a	DHFR
XLOC_211306	ssc-miR-34a	TMEM106C
XLOC_211306	ssc-miR-34a	MLX
XLOC_100516	ssc-miR-328	NDUFA7
XLOC_100516	ssc-miR-328	RPS6KA1
XLOC_100516	ssc-miR-328	PLOD1
XLOC_100516	ssc-miR-328	CERS5
XLOC_100516	ssc-miR-328	RPUSD3
XLOC_100516	ssc-miR-328	PIM1
XLOC_100516	ssc-miR-328	FADS2
XLOC_100516	ssc-miR-328	S100A14
XLOC_100516	ssc-miR-328	ENSSSCG00000001341
XLOC_100516	ssc-miR-328	CALR
XLOC_100516	ssc-miR-328	SLC8B1
XLOC_100516	ssc-miR-328	LRRC8A
XLOC_100516	ssc-miR-328	NUMA1
XLOC_100516	ssc-miR-328	SMPD2
XLOC_100516	ssc-miR-328	SEMA4B
XLOC_000695	ssc-miR-885-3p	ETV4
XLOC_000695	ssc-miR-885-3p	TCOF1
XLOC_100516	ssc-miR-149	TCOF1
XLOC_149196	ssc-miR-149	TCOF1
XLOC_149196	ssc-miR-30c-3p	PPP4R4
XLOC_014459	ssc-miR-30c-3p	PPP4R4
ENSSSCG00000032301	ssc-miR-30b-5p	EED
XLOC_149196	ssc-miR-708-5p	PLPPR5
XLOC_149196	ssc-miR-708-5p	SON
XLOC_000695	ssc-miR-708-5p	PLPPR5
XLOC_000695	ssc-miR-708-5p	SON

## Data Availability

The data used to support this study is available on the SRA database: PRJNA688530.

## Supplementary Materials

Supplementary Materials Supplemental Table S1. Primers used for RT-qPCR in this study. Supplemental Table S2. KEGG pathways associated with target genes of DE lncRNAs. Supplemental Table S3. KEGG pathways associated with target genes of DE miRNAs. Supplemental Table S4. KEGG pathway associated with target genes of DE ceRNAs.
