# Supplementary material for: Analysis of lncRNA, miRNA, and mRNA Expression Profiling in Type I IFN and Type II IFN Overexpressed in Porcine Alveolar Macrophages
Source: Int J Genomics. 2021 Jun 16;2021:6666160. doi: 10.1155/2021/6666160 (PMC8225432; doi:10.1155/2021/6666160)
Supplement: Supplementary Materials — Supplemental Table S1. Primers used for RT-qPCR in this study. Supplemental Table S2. KEGG pathways associated with target genes of DE lncRNAs. Supplemental Table S3. KEGG pathways associated with target genes of DE miRNAs. Supplemental Table S4. KEGG pathway associated with target genes of DE ceRNAs. [file 6666160.f1.docx]

Supplemental Table S1. Primers used for RT-qPCR in this study.

| **Primer** | **Sequence (5' to 3')** |
| --- | --- |
| RT-sscmiR885-3p | GTCGTATCCAGTGCAGGGTCCGAGGTATTCGCACTGGATACGACATCCAC |
| QsscmiR885-3p F | AAGAATAGGCAGCGGGGTG |
| QsscmiR885-3p R | GTCGTATCCAGTGCAGGGT |
| RT-sscmiR708-3p | GTCGTATCCAGTGCAGGGTCCGAGGTATTCGCACTGGATACGACTCTAGA |
| QsscmiR708-3p F | AACACGCCAACTAGACTGTGA |
| QsscmiR708-3p R | GTCGTATCCAGTGCAGGGT |
| RT-sscmiR-29c | GTCGTATCCAGTGCAGGGTCCGAGGTATTCGCACTGGATACGACTAACCG |
| QsscmiR29c F | AACACGCTAGCACCATTTGAA |
| QsscmiR29c R | GTCGTATCCAGTGCAGGGT |
| RT-sscmiR491 | GTCGTATCCAGTGCAGGGTCCGAGGTATTCGCACTGGATACGACCCTCAT |
| QsscmiR491 F | AACAAGAGTGGGGAACCCTT |
| QsscmiR491 R | GTCGTATCCAGTGCAGGGT |
| RT-sscmiR365-5p | GTCGTATCCAGTGCAGGGTCCGAGGTATTCGCACTGGATACGACACAGCT |
| QsscmiR365-5p F | AACAAGGAGGGACTTTCAGGG |
| QsscmiR365-5p R | GTCGTATCCAGTGCAGGGT |
| Qssc-U6 F | TCGCTTCGGCAGCACATATAC |
| Qssc-U6 R | CGAATTTGCGTGTCATCCTTGC |
| qPPP4R4 F | CTGTTCGGCTACATGGAGGA |
| qPPP4R4 R | CTGACCAGCACTGAGCAGATA |
| qDENND3 F | TACATGTTCCACGCGTTCCT |
| qDENND3 R | TCCCGTTTATCCTGTCCTCCT |
| qBATF2 F | CTGCTGACTGGGATGGACTC |
| qBATF2 R | AGGGATGGGAAGGGAAGCAT |
| qPAG1 F | CTGCGTCCCAAAAACACGAAG |
| qPAG1 R | AAATGCTGGCGATGTGTTCC |
| qZC3HAV1 F | TCGTTACCTAGAAGCCCGGA |
| qZC3HAV1 R | GTCAGGTGCAGAACCATTGC |
| ENSSSCG00000036096F | TGGAATCAGAGTGTGTGGGAAG |
| ENSSSCG00000036096R | TCAGGCTGATGGCAATTGAG |
| XLOC_047290F | ATGCAGCTGTTGGCCAATAG |
| XLOC_047290R | TTAAAGGGTCCAGCATTGCC |
| XLOC_039547F | TGTAAACGGATGTGGGATGGAG |
| XLOC_039547R | TACGGTCAAAGCTCCCGAAG |
| XLOC_045711F | GTGGCTCAGCGGTTAATGAATC |
| XLOC_045711R | ATCCTTAACCCACTGAGCAAGG |
| XLOC_149196F | TGTCTTGCCGGATCATTTGC |
| XLOC_149196R | AACATGTCTGCACTCGAAGG |
| XLOC_149269F | ATGCCTTTGCCTTGCTGATG |
| XLOC_149269R | TTGTCACACCTACCAATGCC |
| XLOC_151419F | AGTTCACTGATGGCTTTGCG |
| XLOC_151419R | AGGCCATTCAAAGGAGCATG |
| XLOC_161309F | CTCTTTACACCTCTGCAAACGG |
| XLOC_161309R | ACCTTCCCTTGCATGTCTATGG |
| qGAPDH-F | ACATGGCCTCCAAGGAGTAAGA |
| qGAPDH-R | GATCGAGTTGGGGCTGTGACT |

Supplemental Table S2. KEGG pathways associated with target genes of DE lncRNAs.

| KEGG pathway | P-value | qvalue | gene_name |
| --- | --- | --- | --- |
| Toll-like receptor signaling pathway | 0.0007 | 0.0534 | CXCL11,CD80,B7-1,IFN-ALPHA-9,PIK3CG,p120-PI3K,IFNAR2,FOS,c-fos,CXCL11,CXCL9,MIG,IFNAR1,IFN-R-1,CXCL10 |
| Influenza A | 0.0025 | 0.1362 | JAK1,IFNG,IFNGR2,IFN-gR2,PIK3CG,p120-PI3K,ICAM-1,ICAM1,IFNAR2,IFN-ALPHA-9,NXF1,IFNAR1,IFN-R-1,CXCL10 |
| Jak-STAT signaling pathway | 0.0069 | 0.2485 | JAK1,IFN-OMEGA-3,IFNGR2,IFN-gR2,PIK3CG,p120-PI3K,IFNAR2,IFN-ALPHA-9,IFNG,IFNAR1,IFN-R-1,SPRY2 |
| Natural killer cell mediated cytotoxicity | 0.0100 | 0.2701 | ICAM-1,ICAM1,IFNGR2,IFN-gR2,PIK3CG,p120-PI3K,IFNAR1,IFN-R-1,IFN-ALPHA-9,IFNAR2,PAK1,IFNG |
| p53 signaling pathway | 0.0237 | 0.5442 | CASP3,GADD45G,CD82,CCNB2,SESN1,CDK4,TP53I3,PMAIP1,TN3,FAS,RFWD2,TSC2,CYCS,CYC,THBS1,BAX,  BAX-ALPHA,CDK4,CASP3,SESN2,ATM,MDM2,SESN3,CCNB1,ATR,CCND3,PERP,GADD45B,MDM4,CCNG1,CCND2 |

Supplemental Table S3. KEGG pathways associated with target genes of DE miRNAs.

| KEGG pathway | P-value | qvalue | gene_name |
| --- | --- | --- | --- |
| NF-kappa B signaling pathway | 0.0258 | 0.8656 | LOC110255300,ENSSSCG00000040849,LBP,ENSSSCG00000037885,BCL10,PLCG1 |
| Bacterial invasion of epithelial cells | 0.0367 | 0.8656 | SHC1,ACTB,CLTB,MAD2L2,SEPTIN9 |
| Salmonella infection | 0.0521 | 0.8656 | LOC110255300,LBP,ACTB,KLC2,MAPK12 |
| Inflammatory bowel disease (IBD) | 0.0697 | 0.8656 | LOC110255300,ENSSSCG00000037885,IL21R,RORC |
| Rap1 signaling pathway | 0.0716 | 0.8656 | ENSSSCG00000037885,PLCG1,ACTB,MAPK12,TLN2,PDGFRB,FGFR4,KDR,RALGDS |
| Intestinal immune network for IgA production | 0.0982 | 0.8656 | ENSSSCG00000040849,ENSSSCG00000037885,ITGB7 |
| T cell receptor signaling pathway | 0.1244 | 0.8656 | ENSSSCG00000037885,BCL10,PLCG1,MAPK12,PTPN6,BCL10 |
| Natural killer cell mediated cytotoxicity | 0.1278 | 0.8656 | ENSSSCG00000040849,PLCG1,SHC1,PTPN6,SHC1,SH3BP2 |
| NOD-like receptor signaling pathway | 0.1284 | 0.8656 | LOC110255300,MAPK12,NOD1 |

Supplemental Table S4. KEGG pathway associated with target genes of DE ceRNAs.

| KEGG pathway | P-value | qvalue | gene_name |
| --- | --- | --- | --- |
| Non-alcoholic fatty liver disease (NAFLD) | 0.014370508 | 0.186816604 | MLX,NDUFS6,NDUFA7 |
| Huntington" "s disease | 0.022401538 | 0.232975995 | CLTB,NDUFS6,NDUFA7 |
| Parkinson" "s disease | 0.082337736 | 0.238034122 | NDUFS6,NDUFA7 |
| NOD-like receptor signaling pathway | 0.147158654 | 0.283416667 | NOD1 |
| mTOR signaling pathway | 0.173400648 | 0.290865604 | RPS6KA1 |
| PPAR signaling pathway | 0.193819541 | 0.296429886 | FADS2 |
| Jak-STAT signaling pathway | 0.382294548 | 0.42296418 | PIM1 |
